# Bourbon Virus Transmission, New York, USA

**DOI:** 10.3201/eid2901.220283

**Published:** 2023-01

**Authors:** Alan P. Dupuis, Melissa A. Prusinski, Collin O’Connor, Joseph G. Maffei, Cheri A. Koetzner, Tela E. Zembsch, Steven D. Zink, Alexis L. White, Michael P. Santoriello, Christopher L. Romano, Guang Xu, Fumiko Ribbe, Scott R. Campbell, Stephen M. Rich, P. Bryon Backenson, Laura D. Kramer, Alexander T. Ciota

**Affiliations:** New York State Department of Health, Slingerlands, New York, USA (A.P. Dupuis II, J.G. Maffei, C.A. Koetzner, S.D. Zink, L.D. Kramer, A.T. Ciota);; New York State Department of Health, Albany, New York, USA (M.A. Prusinski, C. O’Connor, T.E. Zembsch, P.B. Backenson);; Suffolk County Department of Health Services, Yaphank, New York, USA (A.L. White, M.P. Santoriello, C.L. Romano, S.R. Campbell);; University of Massachusetts, Amherst, Massachusetts, USA (G. Xu, F. Ribbe, S.M. Rich);; State University of New York at Albany School of Public Health, Albany (L.D. Kramer, A.T. Ciota)

**Keywords:** Bourbon virus, viruses, BRBV, ticks, white-tailed deer, vector-borne infections, arbovirus, zoonoses, *Amblyomma americanum*, lone star tick, surveillance, Suffolk County, New York, United States, *Suggested citation for this article*: Dupuis II AP, Prusinski MA, O’Connor C, Maffei JG, Koetzner CA, Zembsch TE, et al. Bourbon virus transmission, New York, USA. Emerg Infect Dis. 2023 Jan [*date cited*]. https://doi.org/10.3201/eid2901.220283

## Abstract

In July 2019, Bourbon virus RNA was detected in an *Amblyomma americanum* tick removed from a resident of Long Island, New York, USA. Tick infection and white-tailed deer (*Odocoileus virginianus*) serosurvey results demonstrate active transmission in New York, especially Suffolk County, emphasizing a need for surveillance anywhere *A. americanum* ticks are reported.

Bourbon virus (BRBV; genus *Thogotovirus*, family Orthomyxoviridae) is a suspected tickborne human pathogen isolated in 2014 from a patient residing in Bourbon County, Kansas, USA (*1*). BRBV is closely related to Oz virus, which was isolated from *Amblyomma testudinarium* ticks in Japan (*2*,*3*). Since the initial discovery of BRBV, human cases have been identified in Kansas, Missouri, and Oklahoma (*4*). The *Amblyomma americanum* lone star tick has been identified as the likely vector of BRBV transmission and maintenance (*5*,*6*). Small and medium-sized mammals and ground-dwelling birds such as wild turkeys (*Meleagris gallopavo*) are hosts for the immature ticks. Adults feed on large mammals, such as coyotes (*Canis latrans*) and white-tailed deer (*Odocoileus virginianus*). All 3 active developmental stages of the tick will bite humans (*7*). Virus detection in ticks and serologic evidence in mammalian hosts, including white-tailed deer, have been demonstrated in Missouri, Kansas, and North Carolina (*6*,*8*–*10*).

## The Study

In July 2019, New York State Department of Health (NYSDOH) epidemiologists were notified that BRBV RNA was detected in an individual, partially engorged female *A. americanum* tick removed from a Long Island, New York, resident. Comprehensive testing performed through the University of Massachusetts TickReport service (https://www.tickreport.com) revealed the tick was also positive for *Ehrlichia ewingii *bacteria. Notes on the tick submission form indicated the person was experiencing fever, chills, and fatigue; officials with NYSDOH and Suffolk County Department of Health Services (SCDHS) attempted to contact the resident for a follow-up investigation. No additional information was provided, and no blood samples were available to assess potential infection with BRBV.

In 2016, NYSDOH and SCDHS initiated active tick surveillance targeting *A. americanum* ticks for BRBV and Heartland virus (HRTV). HRTV-infected ticks and seropositive deer were detected on Long Island in 2018 and reported in 2021 (*11*). We used standardized flag sampling for the collection of host-seeking *A. americanum* ticks on public lands in Suffolk County. During 2016–2020, a total of 1,265 pools, representing 4,189 adults, 7,227 nymphs, and 97 larvae, tested negative for BRBV RNA by real-time reverse transcription PCR using an in-house multiplex assay to detect HRTV and BRBV (*11*). The BRBV primers for this assay were designed based on the St. Louis strain (GenBank accession no. MK453528) (*12*). During 2021, we expanded sampling for *A. americanum* ticks on Long Island to collect a greater number of ticks from more locations, and we modified molecular detection protocols to use BRBV-specific primers developed at TickReport (Table 1). We designed BRBV-specific primers based on the original virus strain deposited in GenBank (accession no. KU708254) (*13*). We collected a total of 1,058 pools, consisting of 4,406 adults (460 pools) and 9,972 nymphs (598 pools) from 12 sites in Suffolk County, New York. Pool sizes ranged from 1–10 adults and 5–20 nymphs. We detected BRBV RNA in 5 pools of unengorged nymphs. We collected positive pools at 1 site in Smithtown, New York (n = 3), on May 3, 2021, and 2 sites, 1 positive pool each, in Brookhaven, New York, on June 9 and July 8, 2021. We isolated infectious virus from all BRBV RNA-positive tick pools after incubation on Vero cells (ATCC, Manassas, VA, USA). We confirmed that the isolates were BRBV by real-time reverse transcription PCR.

**Table 1:** Primer/probe sets for detection of Bourbon virus RNA in New York, NY, USA*

Name	Gene target	Sequence, 5′ → 3′
BRBV F†	Polymerase subunit, PB1	AACCGGCCAATAGGG
BRBV R	Polymerase subunit, PB1	TGCCAGTTGGGTAGC
BRBV PROBE_5Cy5		/5Cy5/ATGGAGCTG/TAO/CTTTCACTACC/3IAbRQSp/
Bourbon_virus_F1‡	Polymerase subunit, PB1	ATTGCTACTCCGTCCATGTTAGTAAG
Bourbon_virus_R1	Polymerase subunit, PB1	CCAGAACTTGGTAGACATTCCAATAAG
Bourbon_virus_P1_HEX probe		/5HEX/CCCTTGCTG/ZEN/CATCTTCCACCACTTTCACAA/3IABkFQ/

*BRBV, Bourbon virus; F, forward; P, probe; PB1, polymerase basic 1; R, reverse. †Primer/probe set developed at Wadsworth Center, New York State Department of Health, based on Bourbon virus (St. Louis strain) (GenBank accession no. MK453528). ‡Primer/probe set developed at TickReport (https://www.tickreport) based on Bourbon virus (original strain) (GenBank accession no. KU708254)

**Figure 1:**
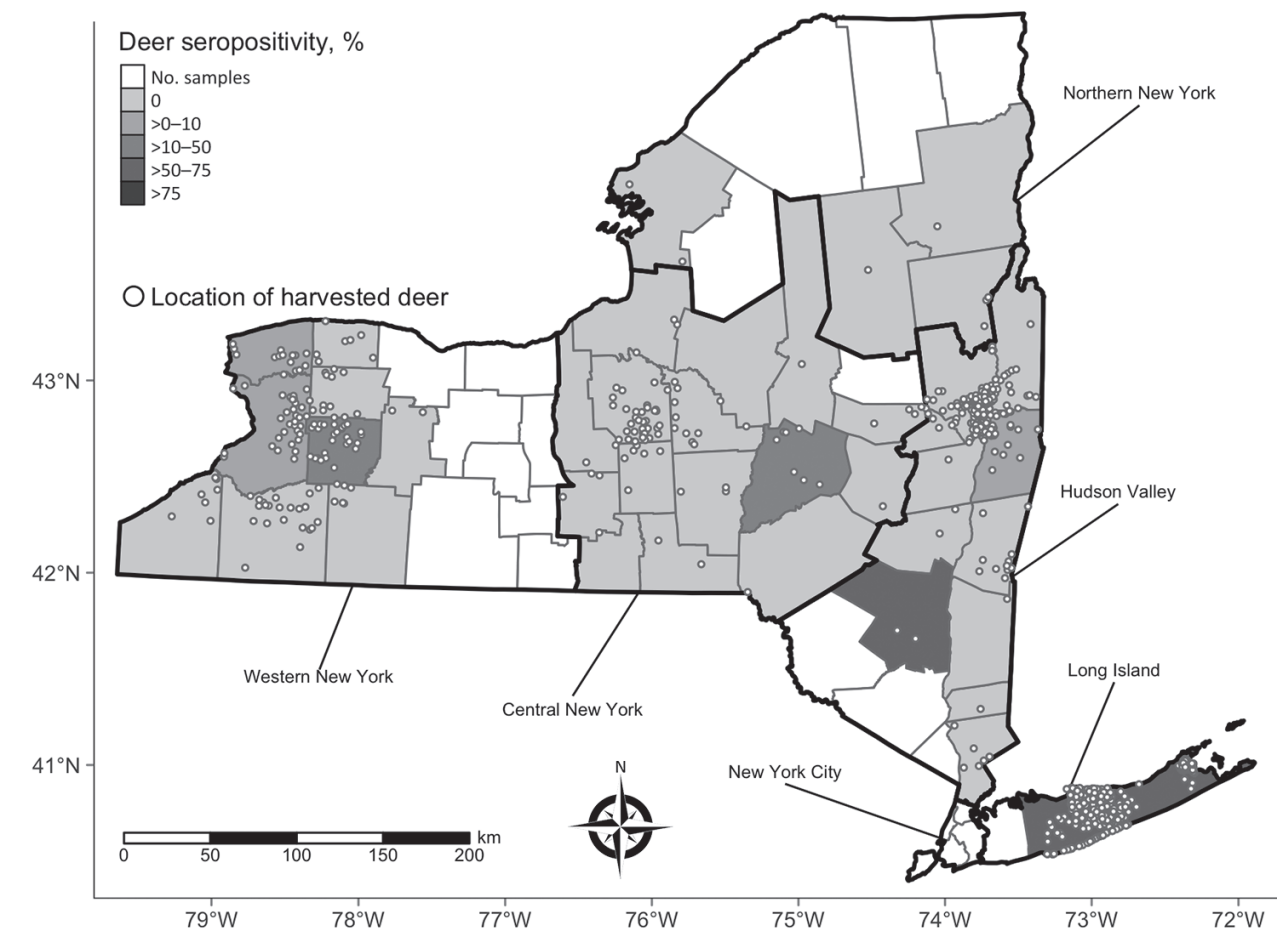
Sampling of hunter-harvested white-tailed deer blood and Bourbon virus seropositivity by county, New York, NY, USA. Locations (open circles) of harvested deer are randomly jittered within townships to avoid overplotting.

**Figure 2:**
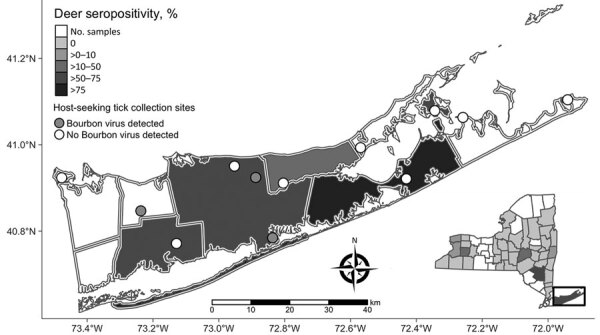
Suffolk County tick collection sites for study of Bourbon virus seropositivity, New York, NY, USA. Circles within townships indicate tick collection sites. Open circles are sites with no evidence of BRBV. Gray circles represent approximate locations of BRBV-positive tick pools. Shading indicates BRBV seroprevalence; darker shades represent higher rates. Inset map shows location of Suffolk County in New York.

Serologic testing of hunter-harvested white-tailed deer blood submitted for arbovirus serosurveys has been conducted in New York since 2007 using plaque reduction neutralization tests, as described (*14*). BRBV was included in these assays starting in 2019 for deer harvested in Suffolk County and the Hudson Valley Region and 2020 for deer harvested in central and western New York. We screened a total of 881 serum samples at 1:20 for the presence of neutralizing antibodies to BRBV (Table 2; Figure 1). We serially diluted samples testing positive for endpoint titers. Statewide, 37.7% of the deer were seropositive; 89.2% of the seropositive deer had titers >20. The seropositivity was 66.5% for deer harvested in Suffolk County (Table 2; Figures 1, 2). Seroprevalence was lower in western New York (3.8%), the Hudson Valley (1.7%), and central New York (1.2%). We did not detect BRBV neutralizing antibodies in 7 deer harvested in the northern New York region (Table 2; Figure 1). We tested *A. americanum* ticks (n = 1,641) collected from 145 deer harvested from Suffolk County for BRBV RNA; the virus was not detected.

**Table 2:** Plaque reduction neutralization test results for Bourbon virus in white-tailed deer specimen samples, New York, NY, USA

Region	Years sampled	No. samples	No. (%) positive
Northern New York	2020, 2021	7	0
Western New York	2020, 2021	132	5 (3.8)
Central New York	2019–2021	80	1 (1.2)
Hudson Valley	2019–2021	176	3 (1.7)
Long Island*	2019–2021	486	323 (66.5)
Brookhaven	2019–2021	291	199 (68.4)
Islip	2021	15	9 (60.0)
Riverhead	2019, 2020	3	1 (33.3)
Shelter Island	2019, 2020	140	85 (60.7)
Southampton	2019, 2020	4	4 (100.0)
Fire Island†	2020	33	25 (75.8)

*Townships are listed for Suffolk County, Long Island. †Fire Island occupies 2 townships but is treated as a single entity for this study.

## Conclusion

Isolation of BRBV from the local tick population and high seropositivity in hunter-harvested white-tailed deer demonstrated active transmission throughout Suffolk County, New York, since 2019. In addition, serologic evidence suggests the virus is present in other regions of New York. Consistent with previous BRBV field studies and the recent discovery of a closely related virus transmitted by *Amblyomma* species ticks in Japan, *A. americanum* ticks were implicated in local transmission of BRBV (*2*,*6*). Tick minimal infection rates were 0%–0.77%. It is unclear whether the unengorged nymphs had acquired the virus as larvae feeding on viremic hosts, through co-feeding transmission, or transovarially. These routes of transmission have been demonstrated in laboratory studies (*5*). BRBV was not detected in adult ticks tested during surveillance despite high numbers collected. Considering the overlap of adult and nymphal tick activity on Long Island, future surveillance campaigns should study the effect of phenology on BRBV transmission.

Of interest, of the 5 BRBV positive pools detected by the TickReport primer set, 1 pool was positive with the primer set used during previous surveillance seasons despite similar assay limits of detection; this result suggested genetic differences in the primer target regions. We plan to conduct phylogenetic analyses and in-vitro growth characteristic studies.

White-tailed deer are a sensitive sentinel model for many arboviruses because of their overall abundance and distribution, small home ranges, and the frequency on which they are fed upon by ticks and other hematophagous arthropods (*14*,*15*). Seroprevalence in Suffolk County deer (66.5%) was higher than that reported in North Carolina deer (56%), but lower than deer harvested in Missouri (86%) (*8*,*9*). BRBV seroprevalence rates of white-tailed deer harvested from various areas in Suffolk County (Table 2) were similar to Oz virus seroprevalence rates (30.0%–73.7%) in wild sika deer (*Cervus nippon*) in Japan sampled from prefectures located near the initial detection of the virus (*3*). The lower seroprevalence in regions of New York outside of Long Island can be attributed to fewer established populations of *A. americanum* ticks or incidental transmission by bird-dispersed immatures originating from established regions. To date, no competent vertebrate host, including deer, has been implicated in BRBV amplification.

Our findings emphasize the need to include emerging pathogens such as BRBV and HRTV in surveillance programs wherever lone star ticks are distributed. Clinicians outside of the midwestern United States should be aware of the potential for human disease. It is unclear if the symptoms of the person who removed the BRBV-positive tick were the result of potential infection with BRBV, *Ehrlichia ewingii*, or an unrelated etiology, because patient blood samples were not available. Considering the overlapping symptomologies of BRBV (fever, fatigue, loss of appetite, thrombocytopenia, and leukopenia) with other tickborne infections, including ehrlichiosis and Heartland virus disease, diagnosis is difficult without specific testing. Currently, testing is only available at the Centers for Disease Control and Prevention and a few state health laboratories. Providers should request BRBV and HRTV testing for patients with history of tick exposure or travel to regions where *A. americanum* ticks are reported and who are displaying clinical symptoms, including leukopenia and thrombocytopenia, that do not respond with antimicrobial treatment.

## References

[1] Kosoy OI, Lambert AJ, Hawkinson DJ, Pastula DM, Goldsmith CS, Hunt DC, et al. Novel thogotovirus associated with febrile illness and death, United States, 2014. Emerg Infect Dis. 2015;21:760–4. 10.3201/eid2105.150150 25899080 PMC4412252

[2] Ejiri H, Lim CK, Isawa H, Fujita R, Murota K, Sato T, et al. Characterization of a novel thogotovirus isolated from *Amblyomma testudinarium* ticks in Ehime, Japan: A significant phylogenetic relationship to Bourbon virus. Virus Res. 2018;249:57–65. 10.1016/j.virusres.2018.03.004 29548745

[3] Tran NTB, Shimoda H, Ishijima K, Yonemitsu K, Minami S, Kuroda Y, et al.; Supriyono. Supriyono. Zoonotic infection with Oz virus, a novel thogotovirus. Emerg Infect Dis. 2022;28:436–9. 10.3201/eid2802.211270 35075999 PMC8798690

[4] Fuchs J, Straub T, Seidl M, Kochs G. Essential role of interferon response in containing human pathogenic Bourbon virus. Emerg Infect Dis. 2019;25:1304–13. 10.3201/eid2507.181062 31211667 PMC6590733

[5] Godsey MS Jr, Rose D, Burkhalter KL, Breuner N, Bosco-Lauth AM, Kosoy OI, et al. Experimental infection of *Amblyomma americanum* (Acari: Ixodidae) with Bourbon virus (Orthomyxoviridae: Thogotovirus). J Med Entomol. 2021;58:873–9. 10.1093/jme/tjaa191 33710315 PMC7955107

[6] Savage HM, Burkhalter KL, Godsey MS Jr, Panella NA, Ashley DC, Nicholson WL, et al. Bourbon virus in field-collected ticks, Missouri, USA. Emerg Infect Dis. 2017;23:2017–22. 10.3201/eid2312.170532 29148395 PMC5708220

[7] Means RG, White DJ. New distribution records of *Amblyomma americanum* (L.) (Acari: Ixodidae) in New York State. J Vector Ecol. 1997;22:133–45. 9491364

[8] Jackson KC, Gidlewski T, Root JJ, Bosco-Lauth AM, Lash RR, Harmon JR, et al. Bourbon virus in wild and domestic animals, Missouri, USA, 2012–2013. Emerg Infect Dis. 2019;25:1752–3. 10.3201/eid2509.181902 31441752 PMC6711231

[9] Komar N, Hamby N, Palamar MB, Staples JE, Williams C. Indirect evidence of Bourbon virus (*Thogotovirus, Orthomyxoviridae*) infection in North Carolina. N C Med J. 2020;81:214–5. 10.18043/ncm.81.3.214 32366639

[10] Savage HM, Godsey MS Jr, Panella NA, Burkhalter KL, Manford J, Trevino-Garrison IC, et al. Surveillance for tick-borne viruses near the location of a fatal human case of Bourbon virus (family *Orthomyxoviridae*: genus *Thogotovirus*) in eastern Kansas, 2015. J Med Entomol. 2018;55:701–5. 10.1093/jme/tjx251 29365128

[11] Dupuis AP II, Prusinski MA, O’Connor C, Maffei JG, Ngo KA, Koetzner CA, et al. Heartland virus transmission, Suffolk County, New York, USA. Emerg Infect Dis. 2021;27:3128–32. 10.3201/eid2712.211426 34648421 PMC8632170

[12] Bricker TL, Shafiuddin M, Gounder AP, Janowski AB, Zhao G, Williams GD, et al. Therapeutic efficacy of favipiravir against Bourbon virus in mice. PLoS Pathog. 2019;15:e1007790. 10.1371/journal.ppat.1007790 31194854 PMC6564012

[13] Lambert AJ, Velez JO, Brault AC, Calvert AE, Bell-Sakyi L, Bosco-Lauth AM, et al. Molecular, serological and in vitro culture-based characterization of Bourbon virus, a newly described human pathogen of the genus *Thogotovirus.* J Clin Virol. 2015;73:127–32. 10.1016/j.jcv.2015.10.021 26609638 PMC5683172

[14] Dupuis AP, Prusinski MA, Russell A, O’Connor C, Maffei JG, Oliver J, et al. Serologic survey of mosquito-borne viruses in hunter-harvested white-tailed deer (*Odocoileus virginianus*), New York state. Am J Trop Med Hyg. 2020;104:593–603. 10.4269/ajtmh.20-1090 33350367 PMC7866319

[15] Clarke LL, Ruder MG, Mead DG, Howerth EW. Heartland virus exposure in white-tailed deer in the southeastern United States, 2001–2015. Am J Trop Med Hyg. 2018;99:1346–9. 10.4269/ajtmh.18-0555 30255829 PMC6221220

